# Personalized peer-comparison feedback and its effect on emergency medicine resident ultrasound scan numbers

**DOI:** 10.1186/2036-7902-6-1

**Published:** 2014-01-14

**Authors:** Dorothea Hempel, Emanuele Pivetta, Heidi H Kimberly

**Affiliations:** 1 Harvard Affiliated Emergency Medicine Residency, Department of Emergency Medicine, Brigham and Women’s Hospital/Massachusetts General Hospital, 75 Francis St, Boston, MA 02115, USA

## Abstract

**Background:**

Clinician-performed ultrasound has become a widely utilized tool in emergency medicine and is a mandatory component of the residency curricula. We aimed to assess the effect of personalized peer-comparison feedback on the number of ultrasound scans performed by emergency medicine residents.

**Findings:**

A personalized peer-comparison feedback was performed by sending 44 emergency medicine residents a document including personally identified scan numbers and class averages. The number of ultrasound scans per clinical shift for a 3-month period before and after the feedback intervention was calculated. The average number of ultrasound exams per shift improved from 0.39 scans/shift before to 0.61 scans/shift after feedback (*p* = 0.04). Among the second year residents, the scans/shift ratio improved from 0.35 to 0.87 (*p* = 0.07); for third year residents, from 0.51 to 0.58 (*p* = 0.46); and from 0.33 to 0.41 (*p* = 0.21) for the fourth year residents before and after the intervention, respectively.

**Conclusions:**

A personalized peer-comparison feedback provided to emergency medicine residents resulted in increased ultrasound scan numbers per clinical shift. Incorporating this method of feedback may help encourage residents to scan more frequently.

## Findings

### Introduction

Clinician-performed ultrasound (CPU) has become a widely used tool in many medical specialties and has been shown to improve patient care and safety [1]. It is considered a skill integral to the practice of emergency medicine (EM) and has been incorporated into EM residency curricula [2]. The training typically consists of a combination of didactics, hands-on scanning, image review, quality assurance and feedback, and observed competency testing. The American College of Emergency Physicians (ACEP) guidelines suggest that residents perform a total number of 150 to 250 ultrasound scans, with a minimum of 25 in each core application during their training [3]. The learning curve for various CPU applications continues to be evaluated, but studies have demonstrated that as the number of performed scans increases, the ability to accurately perform and interpret the images improves [4-7]. As diagnostic accuracy and experience in ultrasound are important both for resident education and ultimately for patient care, feedback interventions that encourage emergency medicine residents to increase their overall scan numbers could be valuable.

The aim of this proof-of-concept study was to evaluate if CPU scan numbers improve after residents are provided with a personalized peer-comparison feedback comparing individual resident ultrasound numbers with their peer group and the peer group average.

## Materials and methods

This study was performed at an academic medical institution with a four-year EM residency program that is combined between two main hospital sites. As part of our ongoing quality assurance process, all ultrasound scans performed by the residents while in the two main emergency departments are entered into a central database after review by one of the ultrasound-fellowship-trained emergency physicians to ensure that the scans meet the minimal criteria. Ultrasounds performed for both teaching and clinical purposes are included. Ultrasounds performed at the community sites or during off-service rotations are not included. Prior to our intervention, residents were provided their individual scan numbers approximately twice per year without comparative data.

Our personalized peer-comparison feedback intervention consisted of a document that was sent by email (using institution addresses) and printed and placed in resident's hospital mailboxes for all residents in post-graduate years PGY-2 through PGY-4. The documents identified each individual's total scan numbers in comparison to their classmates, who were also identified, as well as the class average. Residents were all individually identified and the data was presented in bar-graph format. We did not include PGY-1 residents because of their limited time in the ED due to multiple off-service rotations.

The total scan numbers for each resident were collected from the ultrasound database for a period of 3 months before and 3 months after the peer-comparison feedback intervention. The number of shifts worked in the ED during each 3-month period was retrieved from the shift administration computer system. The data was entered into a spreadsheet (Excel, Microsoft Office, 2007 version).

The scans per shift worked in the ED before and after the intervention were calculated and compared with Wilcoxon signed-rank test for paired data. Statistical significance was defined as *p* value ≤ 0.05. The analysis was performed using STATA software, version 12.0 (StataCorp LP, College Station, TX, USA).

The institutional human research board approval was waived for this study as it consisted of an educational quality initiative.

## Results

Personalized peer-comparison feedback containing the identified total scan numbers of every individual and the class average was sent to 44 emergency medicine residents; 14 second-year residents (six women), 15 third-year residents (nine women) and 15 fourth-year residents (six women).

The mean number of shifts worked in the ED for all residents did not significantly differ in the 3 months prior to the intervention (33.9 shifts/person) and after (36.9 shifts/person), *p* = 0.53. There was a statistically significant difference during pre-and post-intervention in the average total number of ultrasound scans performed by the 44 residents (13.8 scans before and 19.9 after personalized feedback, *p* = 0.05). The average number of ultrasound examinations per shift improved from 0.39 scans/shift before to 0.61 scans/shift after feedback (*p* = 0.04).

**Figure 1:**
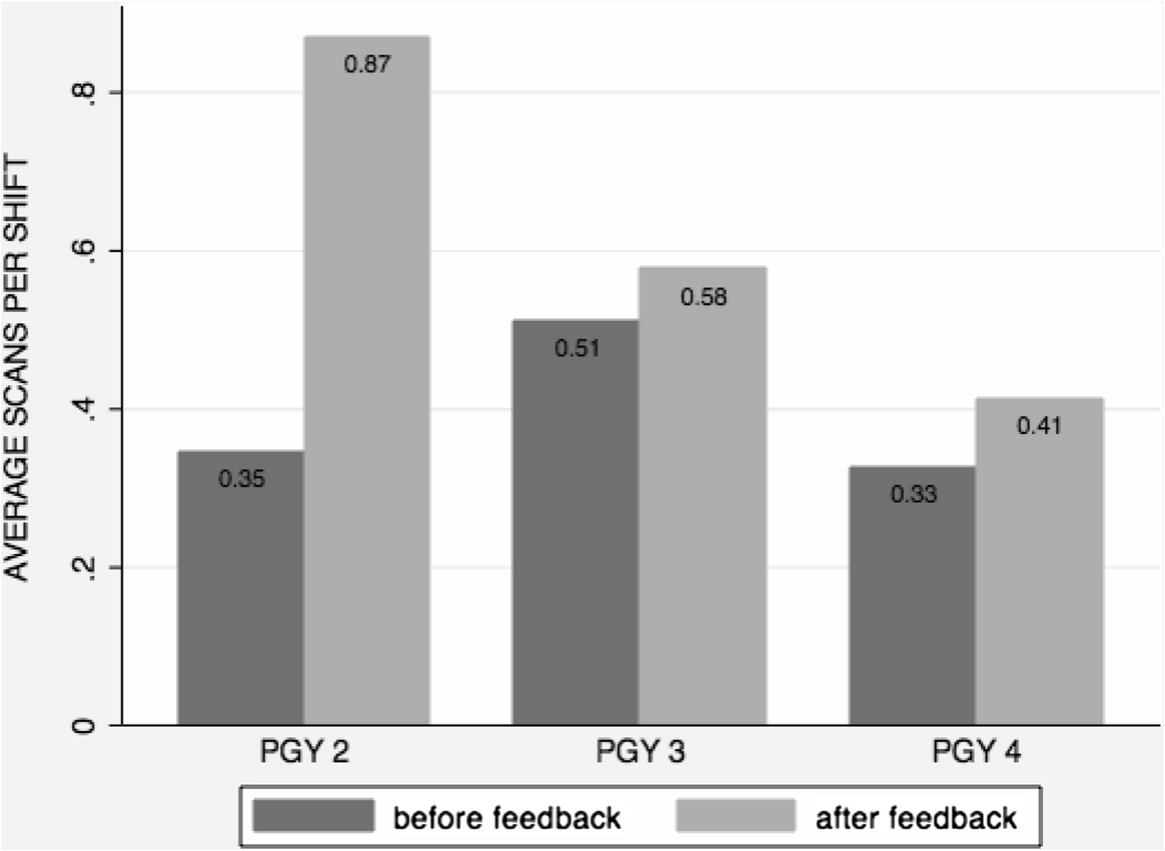
Average ultrasound scans per shift by residency class before and after a personalized feedback intervention.

When evaluated by PGY class, the PGY-2 class had a larger improvement, but no significant difference was observed in the three groups of residents. Among the second year residents, the scans/shift ratio improved from 0.35 to 0.87 (*p* = 0.07); for third year residents, from 0.51 to 0.58 (*p* = 0.46); and from 0.33 to 0.41 (*p* = 0.21) for the fourth year residents before and after the intervention, respectively (see Figure 1). When evaluated by gender, no significant pre- and post-intervention differences were observed. Among male EM residents (*N* = 23), the scans/shift ratio improved from 0.40 to 0.68 (*p* = 0.12) and in women (*N* = 21) from 0.39 to 0.54 (*p* = 0.15).

## Discussion

Performing and interpreting CPU has become an important skill in the daily practice of emergency physicians and is now incorporated into EM residency training [1,2]. As ultrasound and residency educators, we are continually searching for methods to encourage scanning. However, encouraging behavioral changes among adult learners can be challenging. We modeled this study on the success of our department's hand hygiene campaign, which improved significantly after initiating routine reporting of individual providers' hand hygiene statistics. Although there is literature regarding behavior changes in medicine [8], we are not aware of other studies looking at the feedback and behavioral changes regarding CPU. This study evaluated one possible method of feedback, the personalized peer-comparison feedback and its ability to alter behavior. Given that our department already provides other peer-comparison data, we felt that this type of feedback would likely motivate residents without providing excessive peer embarrassment. We found that in the 3-month period after personalized feedback, the mean number of ultrasound scans per shift increased about 20%, from 0.39 to 0.61. This simple intervention could be easily incorporated into residency training and potentially extended to the faculty to encourage increased scanning.

Although not statistically significant for individual residency classes, this approach did appear to be more effective in junior residents. The average scans per shift increased about 20% (from 0.39 to 0.61) for the total cohort but over 50% (from 0.34 to 0.87) among junior residents. A number of potential etiologies are postulated for this finding. It is possible that trainees at different stages respond differently to feedback. Senior residents nearing their end of training may not be as susceptible to peer pressure. Time may also be a factor. Junior residents typically carry fewer patients and may have more time to perform ultrasounds. They also may be more motivated to perform more examinations since ultrasound is a relatively newer skill. Conversely, senior residents may have more advanced clinical judgment and incorporate ultrasound judiciously in a smaller but more clinically significant number of cases. This learning process requires further study, as the ultimate aim of an EM residency ultrasound program is to ensure competency [9].

Even after the intervention, the mean scan numbers remained low (about one scan per shift among junior residents and about half scan among senior residents), suggesting that this specific method of feedback, while useful, may not be sufficient. We did not track the rates of technically limited studies which would have provided information regarding competency. Based on literature evaluating the learning curves of bedside ultrasound, the incidence of technical errors decreases as the scan numbers increase, and there is some suggestion that interpretive skills improve more rapidly than acquisition skills [10]. Thus, we assume that more scans improve competency although the target numbers likely differ by ultrasound application.

We did not specifically evaluate whether the increased ultrasound numbers translated into improved image acquisition or interpretation or whether the scans were clinically relevant or changed patient management. Given the small sample size, we did not investigate if the intervention changed the types of scans performed. Further studies could examine a larger cohort and evaluate if the effect holds over time, assess whether a repeated peer-comparison feedback is still effective, and determine if increased scan numbers lead to improved scanning ability. Ultimately, this type of intervention may prove to be a simple, efficacious, and cost-effective method of feedback and provide incentives for behavioral change during resident education and training.

### Limitations

With only 44 residents, the sample size is small. Using only one training program means that these results have limited external validity. Given the small sample size, we were not able to randomize groups to different methods of feedback and the low power could explain the absence of statistical significance between the residency classes. A multi-centered randomized trial providing personalized peer-comparison feedback would be needed to analyze the effect in different settings (i.e., academic vs. non-academic) and the burden of confounding factors such as gender, age, and experience level, and clinical factors such as number of total patients seen per shift, patient acuity, or attending in charge during shift. We did not attempt to evaluate whether the scans were clinically relevant or changed management although all scans were reviewed for technical acceptability.

## Conclusions

Personalized peer-comparison feedback to emergency medicine residents regarding their ultrasound scan numbers appears to motivate trainees to increase the number of bedside ultrasound studies performed per shift.

## Competing interests

The authors declare that they have no competing interests.

## Authors’ contributions

DH collected data and drafted the manuscript. EP performed the statistical analysis and manuscript editing. HK conceived of the study and participated in its design and coordination. All authors read and approved the final manuscript.

## References

[B1] Moore CL Copel JA Point-of-care ultrasonography N Engl J Med 2011 6 8 749 757 10.1056/NEJMra0909487 21345104

[B2] Lewiss RE Pearl M Nomura JT Baty G Bengiamin R Duprey K Stone M Theodoro D Akhtar S CORD-AEUS: consensus document for the emergency ultrasound Mileston project Acad Emerg Med 2013 6 740 745 10.1111/acem.12164 23859589

[B3] ACEP American College of Emergency Physicians policy statement: emergency ultrasound guidelines 2008 http://www.acep.org/workarea/downloadasset.aspx?id=32878. Accessed 29 June 2012

[B4] Costantino TG Satz WA Stahmer SA Dean AJ Predictors of success in emergency medicine ultrasound education Acad Emerg Med 2003 6 2 180 183 10.1111/j.1553-2712.2003.tb00038.x 12574018

[B5] Gaspari RJ Dickman E Blehar D Learning curve of bedside ultrasound of the gallbladder J Emerg Med 2009 6 1 51 56 10.1016/j.jemermed.2007.10.070 18439787

[B6] Jang TB Ruggeri W Dyne P Kaji AH Learning curve of emergency physicians using emergency bedside sonography for symptomatic first-trimester pregnancy J Ultrasound Med 2010 6 10 1423 1428 20876895 10.7863/jum.2010.29.10.1423

[B7] Jang TB Casey RJ Dyne P Kaji A The learning curve of resident physicians using emergency ultrasonography for obstructive uropathy Acad Emerg Med 2010 6 9 1024 1027 10.1111/j.1553-2712.2010.00850.x 20836789

[B8] Grimshaw JM Shirran L Thomas R Mowatt G Fraser C Bero L Grilli R Harvey E Oxman A O'Brien MA Changing provider behavior: an overview of systematic reviews of interventions Med Care 2001 6 8 Suppl 2 II2 II45 11583120

[B9] Jang TB Coates WC Liu YT The competency-based mandate for emergency bedside sonography training and a tale of two residency programs J Ultrasound Med 2012 6 4 515 521 22441907 10.7863/jum.2012.31.4.515

[B10] Jang T Kryder G Sineff S Naunheim R Aubin C Kaji AH The technical errors of physicians learning to perform focused assessment with sonography in trauma Acad Emerg Med 2012 6 1 98 101 10.1111/j.1553-2712.2011.01242.x 22211463

